# The Broad Clinical Spectrum of Disseminated Histoplasmosis in HIV-Infected Patients: A 30 Years’ Experience in French Guiana

**DOI:** 10.3390/jof5040115

**Published:** 2019-12-13

**Authors:** Pierre Couppié, Katarina Herceg, Morgane Bourne-Watrin, Vincent Thomas, Denis Blanchet, Kinan Drak Alsibai, Dominique Louvel, Felix Djossou, Magalie Demar, Romain Blaizot, Antoine Adenis

**Affiliations:** 1 Service de Dermatologie-Vénérologie, Centre Hospitalier de Cayenne, Avenue des Flamboyants, BP 6006, 97300 Cayenne, France; mbournewatrin@yahoo.fr (M.B.-W.); blaizot.romain@gmail.com (R.B.); 2 Equipe EA3593, Ecosystèmes Amazoniens et Pathologie Tropicale, Université de Guyane, 97300 Cayenne, France; katarina.herceg@hotmail.com (K.H.); denis.blanchet@ch-cayenne.fr (D.B.); felix.djossou@ch-cayenne.fr (F.D.); magalie.demar@ch-cayenne.fr (M.D.); antoine.adenis@ch-cayenne.fr (A.A.); 3 Service de Médecine B, Centre Hospitalier de Cayenne, 97300 Cayenne, France; thomasvinc@gmail.com (V.T.); dominique.louvel@ch-cayenne.fr (D.L.); 4 Laboratoire de Parasitologie-Mycologie, Centre Hospitalier de Cayenne, 97300 Cayenne, France; 5 Laboratoire d’Anatomie et Cytologie Pathologique, Centre Hospitalier de Cayenne, 97300 Cayenne, France; kdrak.alsibai@doctor.com; 6 Unité de Maladies Infectieuses et Tropicales, Centre Hospitalier de Cayenne, 97300 Cayenne, France; 7 Centre d’Investigation Clinique Antilles Guyane, Inserm CIC 1424, Centre Hospitalier de Cayenne, 97300 Cayenne, France

**Keywords:** HIV, histoplasmosis, histoplasma, French Guiana, South America

## Abstract

Histoplasmosis is a common but neglected AIDS-defining condition in endemic areas for *Histoplasma capsulatum*. At the advanced stage of HIV infection, the broad spectrum of clinical features may mimic other frequent opportunistic infections such as tuberculosis and makes it difficult for clinicians to diagnose histoplasmosis in a timely manner. Diagnosis of histoplasmosis is difficult and relies on a high index of clinical suspicion along with access to medical mycology facilities with the capacity to implement conventional diagnostic methods (direct examination and culture) in a biosafety level 3 laboratory as well as indirect diagnostic methods (molecular biology, antibody, and antigen detection tools in tissue and body fluids). Time to initiation of effective antifungals has an impact on the patient’s prognosis. The initiation of empirical antifungal treatment should be considered in endemic areas for *Histoplasma capsulatum* and HIV. Here, we report on 30 years of experience in managing HIV-associated histoplasmosis based on a synthesis of clinical findings in French Guiana with considerations regarding the difficulties in determining its differential diagnosis with other opportunistic infections.

## 1. Introduction

Fungal infections are a major cause of opportunistic infections at the advanced stage of HIV infection [1]. *Histoplasma capsulatum* is a thermally dimorphic fungus described worldwide with the Americas being the world hotspot according to current available data [2]. Infection with *Histoplasma capsulatum* is responsible for histoplasmosis, a disease with a broad spectrum of clinical features included in a well described classification of syndromes that can vary from sub-clinical, acute to chronic disease evolutions [3]. Once the host is exposed to *Histoplasma capsulatum*, disease progression and prognosis are mainly driven by the level of the inhaled fungal burden and by innate or acquired defects in the host immune system. According to these parameters, histoplasmosis may be asymptomatic, self-limited, or invasive and life-threatening, notably in immunocompromised hosts [4]. Hence, HIV-associated histoplasmosis (HAH) is a common AIDS-defining condition in endemic areas for *Histoplasma capsulatum*, with the highest incidence among people living with HIV with a CD4 count <150 cells/mm^3^ [1]. Still, in large case series, the median CD4 count is often much lower than <50 cells/mm^3^ at the time of diagnosis, which affects the disease evolution [5,6,7]. Furthermore, at the late stage of HIV infection, the various clinical features of histoplasmosis may mimic, or be associated with, other concomitant opportunistic infections, making it difficult for clinicians to diagnose histoplasmosis in a timely manner [8].

## 2. Objectives of the Study

The objective of this study was to report on our 30-year experience in the management of HAH, based on a synthesis of clinical results in French Guiana, by taking into account the difficulties encountered in establishing a differential diagnosis with other opportunistic infections, and to put this synthesis into perspective with the current knowledge of this disease.

## 3. Methods

We here present a synthesis of our experience of HAH based on data from six studies conducted by our team in French Guiana between 1991 and 2014 and published [8,9,10,11,12,13]. All patients had both positive serology for HIV and at least one microbiological examination showing fungal elements of *Histoplasma* (direct examination and/or histopathological and cytological examinations and/or culture and/or PCR). Table 1, Table 2 and Table 3 present the clinical, medical imaging, and biological data from 349 patients. Photos of the participants were published with written informed consent.

## 4. General Findings

In people living with HIV, the classical classification of histoplasmosis as acute pulmonary, chronic pulmonary, disseminated sub-acute, and disseminated chronic forms is often less relevant. According to our experience and inmost published case series, up to 95% of histoplasmosis cases are disseminated and may vary from subacute to chronic forms in advanced HIV. Miliary pneumopathy with or without extrapulmonary manifestations such as lymphadenopathy, hepatosplenomegaly, abdominal pain, diarrhea, or shock are frequent. Rarely, patients with a higher CD4 count level (>200 cells/mm^3^) may present with a disease that is restricted to the lungs. 

In 10–60% of HAH cases, severe forms have been described, which can rapidly progress to death by septic shock-like presentation, intravascular disseminated coagulation, hemophagocytosis syndrome, concurrent or isolated massive neurological, renal or pulmonary involvement [2]. These situations require the urgent initiation of the lipid formulation of intravenous amphotericin B (when available) given its fungicidal activity on *Histoplasma capsulatum* [5,6,8]. However, most individuals exposed to *Histoplasma capsulatum* present an initially non-severe form with a progressive evolution over several weeks or months. Usually, disease evolution begins with a focal involvement that may clinically predominate on one or more organs. The lungs, lymph nodes, liver, gastrointestinal tract, central nervous system (CNS), and skin are the most frequent isolated disease presentations and can typically be treated with oral itraconazole.

The diagnosis of histoplasmosis is difficult. Clinical symptoms and signs have low specificity. Gold standard methods for diagnosis are culture-based, slow, poorly sensitive, and require medical mycology skills and facilities (biosafety level 3 laboratory) [4]. *Histoplasma* antibody detection is often unreliable in immunocompromised persons living with HIV, but may be useful in association with other diagnostic methods [14,15]. *Histoplasma* antigen detection methods or molecular biology performed in body fluids have good reported performances but are either mostly unavailable outside of the USA or not commercially available, respectively [1,15]. Furthermore, physician awareness and access to effective antifungals are lacking, making histoplasmosis a neglected disease, responsible for numerous avoidable deaths in people living with HIV in high endemic areas, notably in low and middle income countries [16,17,18]. Differential diagnosis is also challenged by the high frequency of concomitant opportunistic infections with tuberculosis, cerebral toxoplasmosis, and pneumocystosis [1,9]. 

The clinical, laboratory, and imaging features of histoplasmosis are very close to those of tuberculosis, notably in people living with advanced HIV infection. Still, we describe simple patterns for the two diseases to help clinicians distinguish between histoplasmosis and tuberculosis, regardless of the CD4 count level [13]. These factors might help physicians initiate early presumptive antifungal therapy before the results of microbiological cultures are available. A switch from the systematic presumptive anti-tuberculosis therapy in suspected tuberculosis-like cases to a presumptive antifungal therapy for suspected histoplasmosis-like cases could be associated with an important decrease in AIDS-related deaths in endemic areas for *Histoplasma capsulatum*, notably across Latin America [2].

## 5. Clinical Findings

Fever (89%) and weight loss (84%) were constant findings in patients with HAH. These proportions are similar with studies conducted in Colombia and the USA [5,19,20]. The median duration of fever before the first clinical examination was one month.

**Figure 1:**
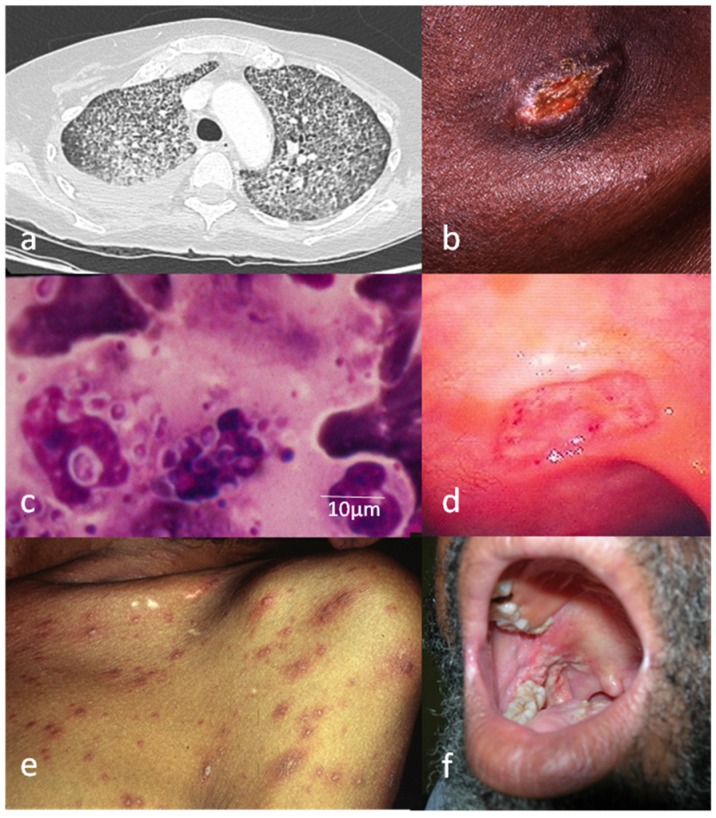
Disseminated histoplasmosis and HIV infection; six characteristic images: (**a**) Pulmonary form; Chest CT-scan: diffuse micronodular opacities of the two lungs in a patient with severe form. (**b**) Lymphadenopathic form with IRIS; supraclavicular lymphadenopathy. (**c**) Direct examination (MGG stain) of bone marrow aspiration: parasitic form of *Histoplasma capsulatum*; small budding yeasts (2–4 µm) surrounded by a pseudo-capsule, intra, and extra-macrophagic. (**d**) Digestive form; colonoscopy: colonic ulceration. (**e**) Cutaneous form; patient with disseminated papules. (**f**) Mucous form: patient with ulcerations of the oral mucosa.

Pulmonary involvement was reported in 62% of cases when considering both data from physical examination and from medical imagery. These results were concordant with most case series, although studies conducted in Colombia and Guatemala reported higher proportions [20,21]. Pulmonary histoplasmosis in patients with HIV characteristically starts with chronic fever and progressive weight loss associated or not with non-productive cough. Cough was the most common pulmonary symptom (39%). Auscultation of the lungs was often normal, but crackles were not uncommon. Respiratory failure with polypnea and dyspnea was frequent (16%) and associated with severe forms. The most frequent pulmonary lesions on the chest x-ray or CT-scan were tiny pulmonary nodules in a miliary distribution (interstitial syndrome) (Figure 1) [9,13,22]. Cavitary lesions, mediastinal involvement, and pleural effusions were uncommon. Hilar or mediastinal lymphadenopathies were rarely reported in chest x-rays (<5%), but frequently reported when performing chest CT-scans (30%). 

In the presence of an interstitial pneumopathy occurring in an HIV-infected patient living in or returning from *Histoplasma capsulatum* endemic areas, three diagnoses must be primarily suspected: pneumocystosis, histoplasmosis, and miliary tuberculosis. Pneumocystis more frequently induces severe dyspnea with a significant decrease in blood oxygen saturation when compared with tuberculosis or histoplasmosis [23]. Hence, when chest CT-scans are unavailable, while waiting for the results of microbiological examinations, the presumptive first line treatment should target pneumocystis. However, in the case of severe respiratory failure, we usually initiate concomitant effective treatment against all three diseases while awaiting results of microbiological examinations. Furthermore, in South-East Asia or in some endemic regions of the Americas, diagnoses of talaromycosis or coccidioidomycosis must be considered. Rarely, other etiologies such as lymphoma, disseminated toxoplasmosis, cryptococcosis, and lymphocytic interstitial pneumonia must be discussed. CT-scans can easily distinguish pneumocystosis from other opportunistic interstitial pneumopathies including histoplasmosis. Typically, a CT-scan in pneumocystosis will show patchy or geographic ground-glass opacities with a smooth septal line thickening also known as “crazy paving” [24]. On the other hand, the presence of hilar or mediastinal lymphadenopathies is an argument against the diagnosis of pneumocystosis. Fungal examinations (direct examination and culture) of the bronchoalveolar lavage fluid after bronchial lavage must be performed to confirm histoplasmosis and search for differential diagnoses or concomitant infections with previously listed pathogens [25]. If available, fungal blood and bone marrow cultures associated with *Histoplasma* antigen detection must be undertaken.

Lymph node involvement was reported in 65% of cases. Superficial lymphadenopathies (cervical, axillary, inguinal) were slightly more common than deep lymphadenopathies (hilar, mediastinal, intra-abdominal) (48% versus 40%).These results were similar to those in studies reported in Colombia, but lower than those conducted in Guatemala and the USA [5,20,21]. 

In the case of lymphadenopathies in HIV infected patients living in endemic areas for *Histoplasma capsulatum*, three diagnoses must be first suspected: tuberculosis, histoplasmosis, and lymphoma. However, if the CD4 count is >200 cells/mm^3^, a diagnosis of histoplasmosis is unlikely. The other differential diagnoses are: HIV lymphadenopathy, Kaposi’s disease, Castleman’s disease, cryptococcosis, and bacillary angiomatosis. It is noteworthy that intra-abdominal lymphadenopathies are frequently associated with involvement of the gastrointestinal tract. In these cases, if lymph node biopsy is not possible, colonoscopy facilitates the diagnosis. Furthermore, immune reconstitution inflammatory syndromes (IRIS) following recent initiation of antiretroviral therapy are frequently associated with histoplasmosis lymphadenitis [26]. 

Lymph node samples (either fine needle aspirates or tissue biopsies) should be assessed thoroughly, both histologically and microbiologically, in order to obtain an accurate diagnosis [27]. Visualization of small yeasts evocative of *Histoplasma capsulatum* on direct examinations stained with MGG may allow a faster diagnosis of histoplasmosis (Figure 1).

Abdominal complaints were frequent with abdominal pain (35%) associated or not with diarrhea (35%) as in studies conducted in the USA (19). Ascites (<5%), lower gastrointestinal bleeding (<5%) or obstruction (<5%) were rare. Hepatomegaly (28%) associated or not with splenomegaly (16%) was frequently reported upon physical examination as in previous studies [5,20]. Liver biopsy is easy to perform and yields a large proportion of positive results. Gastrointestinal mucosa involvement was very frequently reported, similar to a study conducted in Guatemala [21]. Lesions may be observed from the esophagus to the anus, but mainly in the right colon [28]. In our study, colonoscopy with biopsy, when performed, isolated *Histoplasma capsulatumn* in 68% of cases. Endoscopic examination can show, characteristically, small elevated ulcers, either unique, multiple, or diffuse (Figure 1). Differential diagnoses are mainly colonic adenocarcinoma (if unique lesion) or gastrointestinal tuberculosis. Non-ulcerative purple nodules should raise the suspicion of Kaposi’s disease. As gastrointestinal histoplasmosis may be neglected, physicians in endemic areas must be careful when facing abdominal complaints associated or not with fever in people living with HIV. Systematic colonic biopsies sent to medical mycology and pathology should be performed regardless of the presence or absence of any macroscopic mucosal lesions in a symptomatic individual [28,29].

Central nervous system (CNS) involvement occurs in 5–10% of individuals with disseminated histoplasmosis [30]. In our experience, 20% of cases presented signs and symptoms associated with the CNS, notably headache and confusion, similar to previous studies [5,21]. Meningitis, encephalitis, and brain abscess were rare (<5%). In the case of brain abscess, differential diagnosis with cerebral toxoplasmosis is difficult with brain imaging. Failure of presumptive treatment for cerebral toxoplasmosis must lead to a brain biopsy. In the case of meningeal involvement, a progressive meningeal syndrome over several weeks should raise the physician’s suspicion. CSF examinations are not very informative, revealing nonspecific abnormalities with direct examination and fungal culture that are rarely positive [30,31]. In these cases, the demonstration of *Histoplasma capsulatum* in blood, urine, bone marrow, or other samples is crucial.

Mucocutaneous involvement occurs in a wide variation of frequencies according to geographical regions (1–7% in USA and 17–93% in Latin America) [20,32,33,34,35,36,37,38,39], which could be explained by variations in virulence between North and Latin American *Histoplasma* strains [40]. Another explanation would be the difference in access to HIV diagnosis, medical mycology facilities, and the physicians’ awareness regarding the diagnosis of histoplasmosis [6]. Indeed, early diagnosis and treatment rely on the ability to perform either accurate tissue biopsies or fungal blood culture or *Histoplasma* antigen detection in urine and serum. This may prevent the occurrence of mucocutaneous lesions that are probably a late expression of the disease. In our experience, mucocutaneous presentations used to account for 40% of all histoplasmosis cases in the 1990s and are now observed in <10% of cases diagnosed each year. The most common signs were multiple disseminated papules associated with facial acne-like lesions in a context of long- lasting fever (Figure 1) [12]. Plaques, nodules, pustules, or ulcerations, unique or multiple were less frequently observed. Regarding mucous lesions, involvement of the oral cavity was the most frequent; more rarely, lesions can be localized on the lips, pharynx, larynx, and nasal septum [41,42,43]. In the case of a single ulcero-nodular lesion, differential diagnosis must include squamous cell carcinoma. However, the diagnosis of mucocutaneous lesions is simple and rapid because biopsies or even simple MGG-stained smears of these superficial lesions can easily show *Histoplasma* yeasts.

Bone marrow involvement is common. Cytopenias (anemia 89%, neutropenia 40%, and thrombocytopenia 37%) and sometimes pancytopenia, were very frequent. Bone marrow aspiration was very efficient at diagnosing *Histoplasma capsulatum* in culture (78%) and less frequently on direct examination (35%). Histoplasmosis-induced hemophagocytic syndrome (HIHS) may be observed [44]. On physical examination, HIHS is characterized by fever, splenomegaly, and sometimes jaundice. Biological features of HIHS are pancytopenia, hepatic cytolysis (AST > ALT), and an increase in LDH, triglycerides, and ferritin blood levels. Red cell phagocytosis by macrophages on bone marrow examination can determine a diagnosis of HIHS. A comprehensive definition and classification of hemophagocytic lymphohistiocytosis are now available, but little is known regarding HIHS and no specific treatment guidance is available [45,46]. Severe clinical manifestations mimicking septic shock have been reported in around 10% of HAH cases and are probably caused by HIHS [5]. Rapid initiation of lipid formulation of amphotericin B can prevent the onset of shock.

Severity in HAH is difficult to study since no clear, evidence-based definition is available. The Center for Disease Control and Prevention published an extended list of criteria that are not useful for clinicians in distinguishing which patients are or will become severe, in order to introduce effective therapy according to the level of severity observed or expected [47]. To distinguish between severe and non-severe forms, previous prognostic studies have identified the following factors associated with severity: major impairment of the general condition, systolic blood pressure <90mmHg, dyspnea, thrombocytopenia, anemia, renal failure, and high LDH blood level. According to our experience, the LDH blood level may serve as a marker of therapeutic efficacy as blood levels rapidly drop following the initiation of effective antifungal therapy [6]. 

## 6. Atypical Findings

Atypical clinical findings of disseminated histoplasmosis are seldom reported (<5%) in HAH. Ocular lesions are poorly described in people living with HIV and chorioretinal histoplasmosis should be distinguished from other retinitis, notably CMV infection [5]. Pericarditis has been described, but little is known regarding potential cardiac involvement [5]. It is similar for prostatic abscess, sinusitis, muscle and subcutaneous lesions [48,49,50]. Bone or joint involvements are less frequently reported when compared with tuberculosis. However, according to our experience, involvement of the hard palate with perforation following a mucosal form of the oral cavity is the most frequent bone involvement. Adrenal histoplasmosis is frequently reported in non-HIV patients but rarely reported in people living with HIV [51].

An immune reconstitution inflammatory syndrome (IRIS) is sometimes a complication of antiretroviral therapy in HAH patients in the weeks following the initiation of treatment as reported in French Guiana, with a relatively low incidence among patients living with HIV (0.74 cases per 1000 HIV-infected person-years) [26]. Fever may be accompanied by various signs and symptoms: digestive manifestations, lymph node enlargement, hematologic manifestations, and respiratory manifestations. Mucocutaneous, neurological, rheumatological, placental, and ocular findings were less frequent. Severity of cases ranged from 10–50% of HAH with IRIS according to the definition of severe IRIS cases [26].

## 7. Diagnosis

Our experience in French Guiana until 2014 is summarized in Table 3. Positivity rates were highest in tissues rich in immune cells: liver (87%), lymph node (80%), bone marrow (78%), skin and oral mucosa (78%), lower digestive tract (74%), and blood (61%). In 2019, the diagnosis of HAH was based on the detection of *Histoplasma capsulatum* with different laboratory techniques and on different types of samples that were guided by the clinical manifestations. These laboratory techniques are: direct examination stained with MGG, culture on Sabouraud dextrose agar, pathological examination stained with positive acid Schiff (PAS) or Gomori–Grocott, PCR, histoplasma antigen (galactomannan antigen), or serology. Available samples include blood, bone marrow, bronchial lavage fluid, cerebrospinal fluid, urine, and organ biopsies (mainly lymph node, digestive tract, liver, skin, oral mucous). Depending on the hospital resource level, the above-mentioned laboratory techniques and sampling techniques may not be available. Moreover, culture is potentially dangerous, serology is rarely positive in HAH patients, PCR is rarely affordable, and techniques for the detection of histoplasma antigens are not easily available and may cross-react with other fungi. PCRs have been developed in French Guiana, but the lack of commercially available kits, the lack of standardized methods, and the limited number of validation studies are the current limitations in performing histoplasma PCR [52,53,54]. However, in the near future, a simple test allowing a rapid diagnosis on easily obtainable samples (urine, blood) should emerge with the expected availability of a high performance antigen test (Histoplasma galactomannan single-monoclonal-antibody sandwich ELISA) [55,56]. Until these become available, antigenic tests of Aspergillus based on the detection of galactomannan in serum as surrogate marker can be used [57,58,59].

## 8. Conclusions

Nearly forty years after the onset of the HIV pandemic, in most endemic areas for *Histoplasma capsulatum*, physicians managing patients with advanced HIV disease and a non-specific infectious syndrome still rarely suspect histoplasmosis. Over a hundred years after the principal description of histoplasmosis in a case that looked like a miliary tuberculosis, distinguishing between the two diseases remains a challenge for physicians at the patient’s bedside. New *Histoplasma* antigen detection kits are now made available outside the USA for research and hopefully for routine practice in the near future. In Latin America, as in other endemic areas outside the USA, the implementation of educational programs together with increased access to point-of-care diagnostic tools and effective antifungal drugs are required to significantly impact the number of AIDS-related deaths [60].

## Author Contributors

P.C.: literature search, study design, data analysis, data interpretation, writing, figures and tables, revising; K.H.: literature search, study design, data analysis, data interpretation, writing, figures and tables, revising; M.B.-W.: data collection, writing, revising; V.T.: data collection, writing, revising; D.B.: data analysis, figures and tables; K.D.A.: data analysis, figures and tables; D.L.: data analysis, figures and tables; F.D.: data analysis, figures and tables; M.D.: data analysis, figures and tables; R.B.: data collection, writing, revising; A.A.: literature search, study design, data analysis, data interpretation, writing, figures and tables, revising.

## Figures and Tables

**Table 1:** Synthesis of clinical, imaging and endoscopy findings from 349 patients with HIV-associated histoplasmosis in French Guiana [8,9,10,11,12,13].

	% of Patients
**Male (n = 349)**	65
Age > 38 years * (*n* = 349)	52
Concomitant opportunistic infection ** (*n* = 349)	30
**Fever (*n* = 348)**	89
**Weight loss (*n* = 158)**	84
**WHO performance status score > 2 (*n* = 348)**	47
**Systolic blood pressure <90mmHg (*n* = 178)**	18
**Severe forms † (*n* = 349)**	50
Septicemia-like syndrome ‡ (*n* = 349)	12
**Lymphadenopathy (*n* = 349)**	65
Superficial lymph nodes (*n* = 349)	48
Deep lymph nodes § (*n* = 336)	40
**Pleuropulmonary features (*n* = 349)**	62
- **Pulmonary symptoms (*n* = 348)**	48
Cough (*n* = 348)	39
Dyspnea (*n* = 348)	16
Chest pain (*n* = 348)	3
Hemoptysis (*n* = 348)	2
- **Chest (X-ray or CT-scan) (*n* = 318 RX)**	50
Interstitial syndrome (*n* = 318)	41
Alveolar syndrome (*n* = 318)	7
Pleural effusion (*n* = 318)	5
Mediastinal or hilar adenopathies (X-ray) (*n* = 296)	1
Mediastinal or hilar adenopathies (CT) (*n* = 100)	30
**Abdominal features (*n* = 349)**	78
- **Signs and Symptoms (*n* = 348)**	70
Abdominal pain (*n* = 348)	35
Diarrhea (*n* = 348)	35
Hepatomegaly (*n* = 348)	28
Splenomegaly (*n* = 348)	16
Ascites (*n* = 348)	3
Lower gastrointestinal bleeding (*n* = 348)	2
Occlusion/subocclusion (*n* = 348)	1
- **Endoscopy (*n* = 133)**	50
Gastroscopy (*n* = 82)	17
Colonoscopy (*n* = 89)	68
- **Ultrasonography or CT-scan (*n* = 286)**	62
Hepatomegaly (*n* = 286)	37
Splenomegaly (*n* = 286)	29
Adenopathy (*n* = 286)	44
Ascites (*n* = 286)	9
**Neurological symptoms (*n* = 348)**	20
Cognitive impairment and/or confusion (*n* = 348)	8
Headache (*n* = 348)	8
Meningitis/meningoencephalitis (*n* = 348)	3
Brain abscess (*n* = 348)	2
**Muco-cutaneous features (*n* = 349)**	9
Oral lesions ǁ (*n* = 349)	5
Skin lesions ¶ (*n* = 349)	5
Others and atypical features *** (*n* = 349)	10

* Median age = 38 years; ** the main opportunistic infections were esophageal candidiasis 7.5%, tuberculosis 5%, cerebral toxoplasmosis 5% and pneumocystosis 2%; † major impairment of general condition, systolic blood pressure <90mmHg, dyspnea, thrombocytopenia, anemia, renal failure; ‡ Septic shock with intravascular disseminated coagulation, multiorgan failure (kidneys, liver, lungs), rhabdomyolysis, hemophagocytic syndrome; § located either in the chest and/or the abdomen; ǁ usually represented by papule/nodule; ¶ usually represented by ulceration/erosion; *** Pericarditis or pericardial effusion 1–5%, peri anal ulcers 1–5%, sinus involvement <1%, adrenal involvement <1%, bone palate perforation <1%, breast abscess <1%.

**Table 2:** Synthesis of biological findings from 349 patients with HIV-associated histoplasmosis in French Guiana [8,9,10,11,12,13].

	% of Patients
CD4 cell count <200/mm^3^ *	94
CD4 cell count <50/mm^3^	65
Hemoglobin level <11.5 g/dL (*n* = 335)	89
Neutrophil count <1500/mm^3^ (*n* = 332)	40
Platelet count <150,000/mm^3^ (*n* = 334)	37
AST level >34 IU/L (*n* = 332)	73
ALT level >34 IU/L (*n* = 332)	43
Alkaline phosphatase level >150 UI/L (*n* = 302)	48
γ-Glutamyl transpeptidase (GGT) level >50 UI/L (*n* = 303)	76
Lactate dehydrogenase level >300 UI/L (*n* = 305)	71
Creatinine level >100 µmol/L (*n* = 340)	22
C-reactive protein level >100 mg/L (*n* = 309)	26
Ferritinlevel >1000 UI/L (*n* = 164)	57

* Median CD4 cell count = 31/mm^3^.

**Table 3:** Synthesis of mycological, histopathological, and cytological findings from 349 patients with HIV-associated histoplasmosis in French Guiana [8,9,10,11,12,13].

Laboratory Examinations		Number of Examinations (*n*)	% of Patients with a Positive Histoplasma Test
Brochoalveolar lavage			
	DME * +	(*n* = 98)	47
	Culture +	(*n* = 92)	45
Bone marrow aspiration:			
	DME +	(*n* = 208)	35
	Culture +	(*n* = 201)	78
	APC ** +	(*n* = 32)	34
	PCR	(*n* = 40)	58
Mucocutaneous biopsy:			
	DME +	(*n* = 38)	74
	Culture +	(*n* = 29)	55
	APC +	(*n* = 32)	78
	PCR		
Blood culture:			
	DME +	(*n* = 50)	36
	Culture +	(*n* = 62)	61
	PCR	(*n* = 25)	48
Liver biopsy:			
	DME +	(*n* = 65)	26
	Culture +	(*n* = 61)	87
	APC +	(*n* = 75)	51
	PCR +	(*n* = 7)	57
Upper digestive tract-biopsy:			
	DME +	(*n* = 23)	22
	Culture +	(*n* = 22)	41
	APC +	(*n* = 28)	29
Lower digestive tract-biopsy:			
	DME +	(*n* = 55)	56
	Culture +	(*n* = 54)	74
	APC +	(*n* = 59)	73
	PCR +	(*n* = 13)	69
Lymph node biopsy:			
	DME +	(*n* = 55)	42
	Culture +	(*n* = 54)	80
	APC +	(*n* = 63)	62
	PCR +	(*n* = 9)	56
Cerebrospinal fluid:			
	DME +	(*n* = 59)	0
	Culture +	(*n* = 58)	10
	PCR	(*n* = 10)	10
Urine:			
	Culture +	(*n* = 19)	37
Other (sinus, spleen, kidney, lung, ascites fluid):			
	DME +	(*n* = 10)	30
	Culture +	(*n* = 10)	50
	APC +	(*n* = 8)	25
Histoplasma serology			
	Serology positive	(*n* = 58)	10
			
Total:			
	DME +	(*n* = 661)	37
	Culture +	(*n* = 662)	63
	APC +	(*n* = 297)	56
	PCR +	(*n* = 104)	52

* DME: direct microscopic examination (MGG or Gomori–Grocott stains), ** APC: anatomical pathology and cytology (periodic acid Schiff or Gomori–Grocott stains).
